# Assessing the health workforce implications of health policy and programming: how a review of grey literature informed the development of a new impact assessment tool

**DOI:** 10.1186/s12960-017-0252-x

**Published:** 2017-11-09

**Authors:** Andrea Nove, Giorgio Cometto, James Campbell

**Affiliations:** 1 Novametrics Ltd, Derby, United Kingdom; 2 World Health Organization - Health Workforce Department, Geneva, Switzerland

**Keywords:** Human resources for health, Health workforce, Impact assessment, Global Strategy on Human Resources for Health, Health labour market, Global health initiatives, Grey literature

## Abstract

**Background:**

In their adoption of WHA resolution 69.19, World Health Organization Member States requested all bilateral and multilateral initiatives to conduct impact assessments of their funding to human resources for health. The High-Level Commission for Health Employment and Economic Growth similarly proposed that official development assistance for health, education, employment and gender are best aligned to creating decent jobs in the health and social workforce. No standard tools exist for assessing the impact of global health initiatives on the health workforce, but tools exist from other fields. The objectives of this paper are to describe how a review of grey literature informed the development of a draft health workforce impact assessment tool and to introduce the tool.

**Method:**

A search of grey literature yielded 72 examples of impact assessment tools and guidance from a wide variety of fields including gender, health and human rights. These examples were reviewed, and information relevant to the development of a health workforce impact assessment was extracted from them using an inductive process.

**Results:**

A number of good practice principles were identified from the review. These informed the development of a draft health workforce impact assessment tool, based on an established health labour market framework. The tool is designed to be applied before implementation. It consists of a relatively short and focused screening module to be applied to all relevant initiatives, followed by a more in-depth assessment to be applied only to initiatives for which the screening module indicates that significant implications for HRH are anticipated. It thus aims to strike a balance between maximising rigour and minimising administrative burden.

**Conclusion:**

The application of the new tool will help to ensure that health workforce implications are incorporated into global health decision-making processes from the outset and to enhance positive HRH impacts and avoid, minimise or offset negative impacts.

**Electronic supplementary material:**

The online version of this article (10.1186/s12960-017-0252-x) contains supplementary material, which is available to authorized users.

## Background

The attainment of national and global health goals can be achieved only with a health workforce that is adequate in number, distribution, skills, motivation and performance. This fact has been formally recognised by the inclusion of a ‘health worker density and distribution’ indicator within the monitoring framework for the sustainable development goals (SDGs) [1]. In 2016, the World Health Assembly (WHA) adopted *Workforce 2030*: the World Health Organization (WHO) Global Strategy on Human Resources for Health (HRH) [2], which sets out the policy agenda to ensure that the health workforce is adequate, well distributed and fit for purpose and thus allowing for the attainment of the SDGs, while also contributing to economic growth through the creation of qualified employment opportunities in the health sector, as underscored by the UN High Level Commission on Health Employment and Economic Growth [3]. The WHA resolution adopting the Global Strategy on HRH includes three key actions relating to assessing the health workforce implications of health policy and programming:

1. Development partners should coordinate and align their investments in education, employment, health, gender and labour in support of domestic financing aimed at addressing national health workforce priorities.
2. Global health initiatives should ensure that all grants include an assessment of the health workforce implications, leverage national coordination and leadership, and contribute to efficient investment in and effective implementation of national health workforce policies.
3. An assessment should be made of the health workforce implications of technical resolutions brought before the World Health Assembly and WHO regional committees [4].

These provisions in the Global Strategy and the accompanying resolution aim to address challenges that have become apparent over the last decade. For instance, the impact of investments by global health initiatives on HRH is mixed, with results failing to achieve their full potential and unintended but sometimes detrimental effects caused by distortionary incentives and practices [5–7].

Development initiatives may potentially lead to a number of impacts on the health workforce which occur at various levels of the health system and over different time periods (short, medium and long term), many of which can easily be overlooked. Examples include:

- Creation of new jobs, preservation of existing jobs, job losses [8–10]
- Changes to the skill set of the workforce [11]
- Changes to the profile of the workforce, e.g. more or fewer people with disabilities, older workers, women, ethnic minority groups [8, 11]
- Changes to terms and conditions of employment or what is expected of health workers [8–12]
- Displacement effects, e.g. health worker migration [10]
- Increase or reduction in productivity, efficiency, absenteeism [12]
- Increased or decreased motivation to invest domestic resources in the health workforce, or political opposition from HRH stakeholders [13]

This approach is conceptually similar to the Health in All Policies (HiAP) approach, which expects all public policies to take into account the health implications of decisions, and avoid harmful health impacts. This approach recognises that health is one of a number of competing priorities, but maintains that health considerations must always be taken into account when policy decisions are made [14]. Similarly, the WHA resolution relating to the Global HRH Strategy recognises that HRH may not be a central element of a grant, investment or resolution of an international organisation, but the HRH implications should be assessed regardless. This will help to ensure that the implications (if there are any) are taken into account, even if they are unintended or indirect.

The HiAP approach involves the undertaking of systematic health impact assessments [14]. Impact assessment (IA) is a process that prepares evidence for decision-makers concerning the advantages and disadvantages of initiatives, based on an assessment of their potential outcomes and impacts [15, 16]. *Ex ante* IAs aim to identify, predict, evaluate and mitigate the consequences of development proposals *prior to* major decisions and commitments being made [17]. They represent an attempt to provide, in advance, a coherent and transparent analysis of the reasoning that lies behind, and the foreseeable effects of, any proposed policy or intervention, and thus have the potential to improve the quality and/or efficiency of these policies and interventions [18–22].

There were calls for the mainstreaming of ex ante health workforce impact assessments (HWIAs) as long ago as 2002 [23–25]. At that time, the specific benefits of such assessments were seen to be as follows: (i) they would draw the attention of decision-makers to the potential consequences of their decisions for HRH, (ii) they would help steer organisational and financing decisions towards minimising negative effects on the workforce and enhancing positive ones, and (iii) they would help to build up documentation on how HRH are affected by new policy initiatives [23].

Fifteen years later, these issues remain highly relevant to national and international development agendas, yet HWIAs are still not common practice and there are no established tools and techniques for them. If the actions contained within the 2016 WHA resolution are to be implemented systematically, there is a need to develop such tools and techniques. A well-designed and well-implemented HWIA is a positive opportunity to make better decisions [16, 26] as well as being a way to fulfil the requirements of the Global HRH Strategy.

This paper presents the findings of an exploratory analysis of IA tools and guidance developed by the health and other sectors. The aim of this analysis was to locate examples of guidance and tools for IA on a wide range of topics, and thus to inform the development of an HWIA tool, a draft of which is appended to this paper. Reflecting the three key actions highlighted in the WHA resolution, the tool’s audience is expected to be technical, managerial and political decision-makers representing development partners, global health initiatives and the WHO Secretariat.

## Methods

Given the type of materials sought, the search was limited to grey literature. The following search terms were entered into the Google search engine by a researcher based in the UK:

- Ex ante impact assessment (+ WHO, UNFPA, ILO, UNICEF, Global Fund, Gates Foundation, Norad, USAID, AusAID, development grants, development funding, development assistance, checklist, criteria, screening)
- Ex ante assessment (+ workplace, workforce, employment, human resources, skills)
- Ex ante needs assessment
- Ex ante assessment tool
- Impact assessment (+ tool, template, health, gender, disability, age, equality, employment, workplace, workforce, human resources, skills)

The above search strategy yielded hundreds of thousands of results. For each search term, the first six to eight pages of results were reviewed (there were approximately 10 results per page), excluding any sponsored or promoted web pages, until the results became mostly duplicates or irrelevant. This stage of the review consisted of clicking on the URL and assessing (a) whether or not the link included an IA tool, report or guidance, and if so (b) whether or not these had been developed or used by or on behalf of national or local governments (or their agencies), United Nations (UN) agencies, the European Union (EU), the Organisation for Economic Cooperation and Development (OECD), a national health service, a funder of global health initiatives, or the International Association for Impact Assessment (IAIA). If yes, a note was made of the URL so that the documents could be read in more detail later. A snowballing technique was also used; if one of the located documents cited other relevant guidance, tools or reports, these other documents were also sought and added to the review if they met the above criteria. The search was conducted in English, between August and November 2016.

The initial search yielded 51 examples of guidance, tools and reports relating to IAs. The additional snowballing yielded a further 21 documents, bringing the total to 72. Information was located about a wide range of types of IA, as shown in Table 1. The earliest examples were from the late 1990s, and the latest from 2016.

**Table 1:** Summary of search results

Assessing the impact on	Number of positive search results^a^	Types of documents located	Years in which documents published
Equality	13	Guidance, tool, report	2008–2015
Health	11	Guidance, tool, report	1999–2010
Human rights	8	Guidance, tool, report	2001–2014
General	8	Guidance, report	2001–2015
Employment	6	Guidance, report	2000–2016
Regulation	6	Guidance, tool, report	2003–2016
Agriculture and fisheries	2	Guidance, tool, report	2013
Business continuity	2	Tool	Not given
Environment	2	Guidance	1999–2002
Gender	2	Guidance, report	2000–2009
Investment	2	Report	2013
Poverty	2	Guidance, tool	2007
Other	10	Guidance, tool, report	2001–2015

^a^This column adds up to 74 rather than 72 because two documents covered more than one of the listed topics: one covered both equality and human rights and one covered both gender and employment

Out of the 72 documents, 44 were tools or guidance/reports that included a tool. Table 2 shows that most of these originated from the European Union (EU), Organisation for Economic Cooperation and Development (OECD) or the United Kingdom (UK). This is probably partly a function of the search being conducted from the UK, and partly due to the fact that the EU and the OECD were early adopters of IA as part of their approach to developing new policies and programmes [18, 27].

**Table 2:** Country or organisation of origin of the impact assessment tools located

Assessing the impact on	Number of tools	Country or organisation of origin
Equality	13	EU (1), UK (12)
Health	5	Sweden (1), Switzerland (1), UK (3)
Human rights	5	EU (1), Norway (1), UK (1), UN agency (3)
General	2	EU (1), Moldova (1)
Employment	1	EU (1)
Regulation	3	Hungary (1), EU (1), UK (1)
Agriculture and fisheries	2	Malawi (1), multi-country (1)
Business continuity	2	UK (2)
Environment	2	OECD (1), UN agency (1)
Gender	1	EU (1)
Investment	0	–
Poverty	1	OECD (1)
Other	7	EU (1), Finland (1), Ireland (1), UK (2), UN agency (2)

Each of the 72 identified documents was read, and notes made on their contents when these were considered to be potentially relevant to the development of an HWIA tool. Based on these notes and printouts of the tools located during the above search, a draft HWIA tool was developed. The process of extracting information from the identified documents was an inductive one, with themes becoming apparent as the review progressed.

**Fig. 1:**
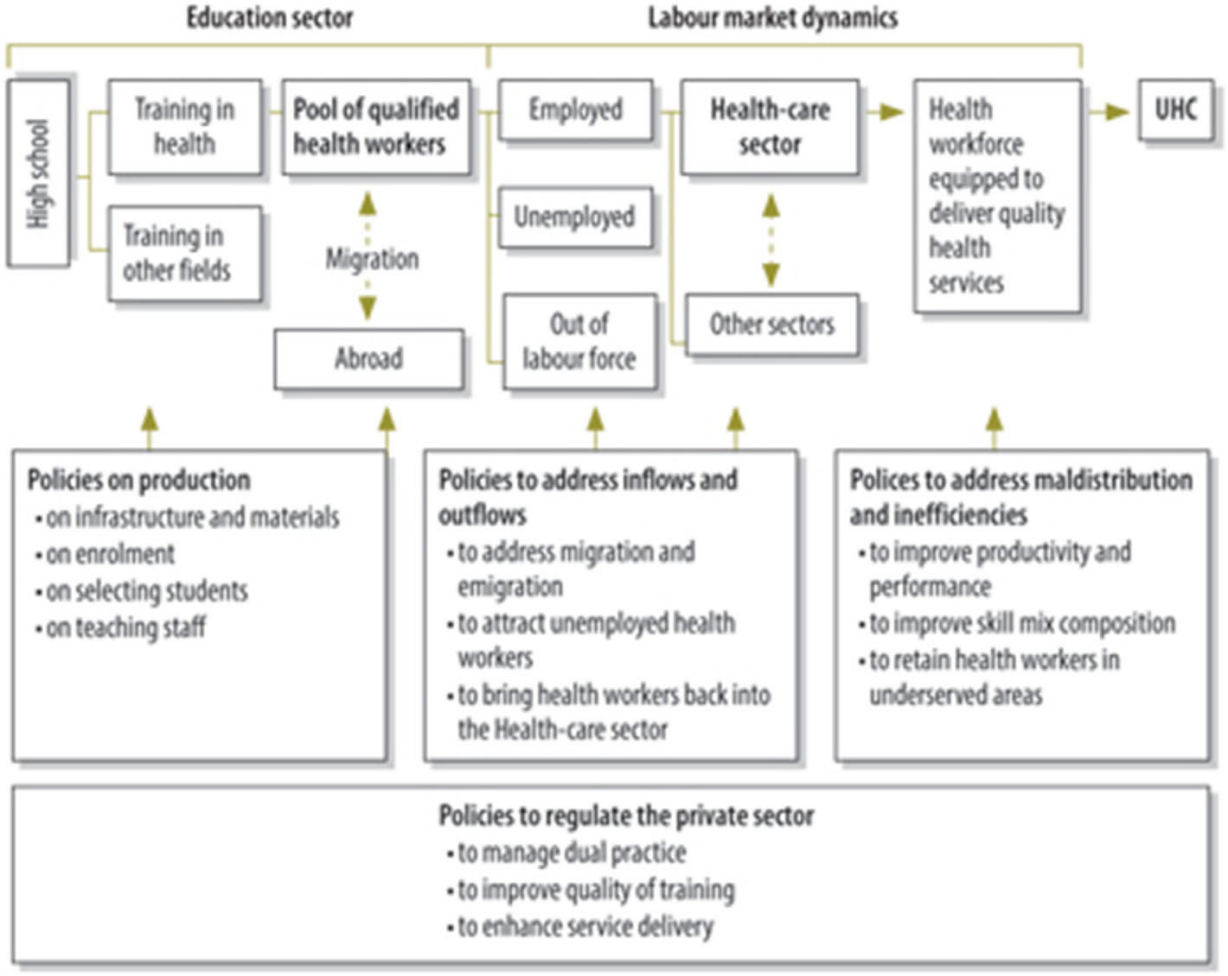
Health labour market framework. Sousa et al. [51]

To ensure that the tool was comprehensive and structured, a number of potentially relevant conceptual frameworks was considered as a basis for the review, including a ‘right to health’ framework [20], a social determinants framework [28], and an equity framework [9]. Given the need to make the tool specific to HRH, the WHO health labour market framework (Fig. 1) was considered to be the most relevant, since it will help to ensure that all aspects of HRH and all relevant ‘policy levers’ are considered during an assessment.

## Findings of the literature review

The review indicated that there is no single model used widely for IAs [8, 29, 30]. A limited amount of literature explains how to carry out an ex ante IA, and there is a particular dearth in the field of international development and low- and middle-income country contexts [30]. Table 1 shows that literature and documentation about health workforce assessments is lacking: none of the documents located via the review related to a health workforce assessment. Nevertheless, the reviewed documents contain a number of common and relevant themes, which could form the basis of guidance for the design and execution of an ex ante HWIA, and these are described in this section.

### General principles

It is helpful for an IA to be based on an established conceptual framework [31, 32], since this helps it to be purposive, comprehensive, focused and relevant. To achieve its objectives fully, an IA should set out the logical reasoning that links the problem or issue that the initiative is designed to address, the underlying drivers of this problem, the objectives of the initiative, and the available policy options to achieve these objectives [33]. To accomplish this, the assessment process must be appropriately resourced [34] and ideally demonstrate the following characteristics:

- Comprehensive and interdisciplinary, i.e. recognising that the initiative being assessed may be determined by a broad range of factors [12, 17, 22, 34–36]
- Evidence-based, with proper documentation of the process as well as the findings and conclusions [26, 29, 34, 37]
- Rigorous and systematic yet practical, efficient and cost-effective, i.e. using methods that are appropriate and proportionate to the issues being investigated and which result in acceptable and feasible solutions [17, 26, 28, 34, 36, 38]
- Equitable, i.e. considerate of how impacts might be different for different groups of people or organisations [22, 28, 30, 35, 36]
- Forward-looking, i.e. considerate of both short- and long-term impacts and implications [28, 36, 37]
- Aligned with relevant strategies and policies [13, 32, 39]

It is common in the literature to consider an IA as an extension of the logical framework (logframe) or causal chain analysis used by many donors [32], i.e. an assessment may consider inputs, outputs, outcomes and impacts (with a strong focus on outcomes and impacts) [12, 40]. However, IAs also present an opportunity to consider the ‘missing middle’, i.e. the processes by which outcomes and impacts are achieved, because they can investigate assumptions, contextual conditions, underlying mechanisms and causal links [8, 12, 31]. Arguably, this is particularly true for ex ante assessments, given that they cannot attempt to measure actual impacts [40].

There is no consensus about whether it is better to use quantitative or qualitative methods to conduct an IA. On the one hand, some practitioners see quantitative methods as more robust and reliable [12, 27, 41] and therefore recommend the use of techniques such as cost-benefit analysis and econometric models [15, 42, 43]. On the other hand, critics of this viewpoint argue that these methods tend to focus on a narrow set of impacts, leave questions unanswered about wider dynamics, and can exclude the perspectives of relevant stakeholders [30]. However, qualitative techniques seem to be less well developed, perhaps indicating a need for more structured ways of analysing impacts in a qualitative way. Some advocate a mixed methods approach, using qualitative information to enrich quantitative data and estimates [30, 40].

### Objectives of an ex ante impact assessment

Two main objectives of an ex ante IA were identified: (1) to ensure that the topic being assessed is explicitly addressed and incorporated into the decision-making process, and to anticipate the likely impacts on it [17, 35], and (2) to generate more positive outcomes, by enhancing the positive impacts and avoiding/minimising/offsetting negative impacts [20, 30, 37].

### Structure and content of an assessment tool

IA tools generally fall into one of the three types: checklists, flowcharts and matrices [30]. Some of the advantages and disadvantages of each type are summarised in Table 3.

**Table 3:** Advantages and disadvantages of types of impact assessment tool

Type	Advantages	Disadvantages
Checklist	Easy to understand and use	May not distinguish between direct and indirect impacts. Does not link action to impact
Flowchart	Links action to impact. Useful for checking for indirect impacts	Can become very complex very quickly
Matrix	Links action to impact	Can be cumbersome. Difficult to address probability of impact occurring

Source: Adapted from Foresti et al. [30]

For the effort involved in an assessment to be proportionate to the scale of the likely impact, many commentators recommend splitting the assessment tool into two sections. The first section is a short screening module which aims to work out whether or not the initiative being assessed will have any impact on the topic being assessed [29, 44]. The screening module can therefore consist mainly of simple ‘tick box’ questions. The second section is used only for those initiatives found at the screening stage to have expected impacts, and consists of a more detailed examination of these [20, 22, 30, 35, 36, 44]. At this stage, extensive use can be made of open questions, with well-chosen examples as prompts [45].

Impacts can be defined as the changes that occur as a result of a policy or intervention [30]. Impacts can be direct or indirect, intended or unintended, positive or negative [12, 19, 28, 31, 37]. Direct impacts may prompt indirect ones, which can be just as important and may represent an important link in the chain of actions to the solution to the identified problem [46]. Further, impacts may occur at different levels [8], so an IA tool should aim to capture impacts of all relevant types and levels. Additionally, IAs often consider risks, i.e. things that might undermine successful implementation of an initiative or unforeseen negative consequences [26, 31, 41, 47].

Many commentators agree that stakeholder mapping is an essential element of an ex ante IA [8, 16, 29, 30, 33, 38], to (a) establish who will be affected by and/or influential over the implementation of the initiative [31, 45] and (b) understand each stakeholder’s attitude to, level of interest in or influence over the initiative so that appropriate and timely engagement with important stakeholders can be planned from the outset, thus maximising the chances of successful implementation [45].

### The process of an impact assessment

IAs represent an opportunity to be transparent about the reasons for decisions and actions and allow other stakeholders to examine the assumptions, logic and evidence underlying resource allocation decisions, and thus have the potential to improve accountability and multi-sectoral working [32, 35]. For this reason, the process is at least as important as the results, and it may encourage policy- and decision-makers to think beyond what may normally be a relatively narrow remit [27]. Embedding IAs within an organisation’s planning and policy cycle is a good way for that organisation to get into the habit of conducting IAs as a matter of routine [29, 34]. If an organisation is already in the habit of conducting IAs for issues other than HRH, it may be possible to incorporate HWIAs into their existing process [20, 35].

An ex ante IA is used to help design and define an intervention [31], which means that the assessment should happen well before implementation so that its recommendations can be taken into account before critical decisions are made [11, 16, 20, 29, 33, 37, 48]. To ensure the assessment is appropriately focused, it should begin with a clear statement of the objectives of the initiative being assessed, an explanation of what problem(s) or issue(s) it is designed to address, and the causes of these problems or issues [15, 18, 20, 33, 49]. A full-scale assessment should ideally be a participatory process, involving an appropriately wide selection of people and organisations who will be interested in and/or affected by the implementation of the initiative being assessed [17, 22, 28, 29, 33–36].

It is good practice to produce and disseminate a report of the assessment, which describes the expected impacts and implications if there are any, and if none is expected, justifies why this conclusion has been reached [33].

## Applying the results of the review to the development of a health workforce impact assessment tool

The established conceptual framework on which the draft HWIA is based is the WHO health labour market framework (see Fig. 1). Three main objectives of an ex ante HWIA were identified, via adaptation of the objectives of IAs in other fields: (1) to ensure that HRH are explicitly addressed and incorporated into the decision-making process, and to anticipate the likely impacts on HRH, (2) to generate more positive outcomes, by enhancing the positive impacts on HRH, avoiding/minimising/offsetting negative impacts on HRH and/or improving efficiency, and (3) to ensure that the HRH implications are not overlooked, especially when they are not obvious.

A distinction is made in the HWIA objectives between HRH *impacts* and *implications*. Impacts can be defined as the effects on the health workforce of the initiative being assessed. Implications come into play when there is a degree of dependency on the health workforce for successful implementation of the initiative, so HRH requirements need to be considered as part of the planning and implementation process. Unlike some of the other topics for which IAs have been carried out, an initiative could have HRH impacts or implications (or both). This indicates that an HWIA tool must be able to capture both, so the draft tool addresses implications in Sections A4 and B1 and impacts in Sections A5 and B2. The concept of implications aligns with the concept of ‘risk’, which many commentators recommend including in an IA—if an initiative is in any way dependent on the health workforce, an IA must consider whether there is a risk that the workforce will be unable or unwilling to contribute to implementing it. In the case of HRH, the risks could include insufficient health workers in the right locations, insufficient skills or equipment to deliver the intervention, perverse or insufficient incentives, low health worker morale or lack of motivation [50].

Table 4 presents the different sections of the draft tool and explains how and why these sections follow the guidance obtained via the literature review. The draft tool itself can be found in Additional file 1.

**Table 4:** Description of the draft tool and rationale for each section

Section of the tool	Technique(s) used	Rationale for inclusion
Introduction	Explanatory text	To explain when, how, why and by whom an assessment should be carried out, and what should happen to the results. To emphasise the multi-sectoral nature of the process and the need for an evidence-based approach to the process.
A1: Details of the initiative being addressed	Open questions	To emphasise the importance of alignment with the relevant strategies and policies.
A2: Contributors to the assessment	Open questions	To help ensure transparency and accountability.
A3: Objectives of the initiative	Open questions with prompts	To help ensure the assessment focuses on these objectives.
A4: Extent to which implementation is dependent on the health workforce	Checklist	To ensure that HRH implications are considered as well as HRH impacts.
A5: Anticipated health workforce impacts	Checklist	To prompt the user to try to anticipate all types of direct and indirect, positive and negative, intended and unintended HRH impacts.
B1: Understanding the initiative’s dependency on the health workforce	Checklist + open questions	To encourage consideration of processes, assumptions and causal links as well as outcomes and impacts. To encourage consideration of different options to address the anticipated impacts and implications.
B2: Understanding the initiative’s impact on the health workforce	Checklist + open questions	
B3: Equity analysis	Checklist + open questions	To ensure consideration of how impacts might be different for different groups.
B4: Stakeholder analysis	Checklist + open questions	To assist the organisation to build in appropriate HRH stakeholder engagement activities to the initiative.
B5: Legal and political considerations	Open questions with prompts	To ensure that legal and political considerations are not overlooked.
B6: Next steps	Checklist + open questions	To encourage action to be taken to address the issues highlighted by the assessment.
B7: Additional comments	Open question	To allow the inclusion of important issues not covered in the earlier sections. To give space to justify a decision not to take action as a result of the assessment.

The draft HWIA tool begins by establishing the objectives of the initiative being assessed, an explanation of what problem(s) or issue(s) it is designed to address, and the causes of these problems or issues (see Section A3 in Additional file 1), which will help to focus the assessment on the appropriate issues.

The draft tool has the desirable characteristics highlighted by the literature review. It recognises that HRH impacts and implications may be determined by a broad range of factors, by not restricting users to focusing only on factors within the health sector. It encourages the collation of supporting evidence and proper documentation of the process as well as simply reporting the findings and conclusions (see, for example, part (b) of Section B1). It is systematic yet practical, using a method that is proportionate to the relevance of HRH to the initiative being assessed (the screening Module (part A) is relatively short and simple to complete so that initiatives with few or no health workforce impacts or implications will not need to conduct a full assessment). It considers how impacts might be different for different groups of people or organisations (see Section B3). It considers both short- and long-term impacts and implications (see, for example, the preamble to Section A5). It includes a stakeholder mapping exercise (see Section B4). There are detailed instructions about the process (see the first two pages of the tool), accountability is promoted by the requirement to record the names of those carrying out the HWIA in Section A2, and those completing a full review are prompted to start an action plan in Section B6. Finally, the tool is aligned with relevant strategies and policies such as the SDGs and the Global Strategy on HRH.

As an ex ante IA, the draft tool also takes advantage of the opportunity to consider the processes by which impacts are achieved as well as the impacts themselves. It does this by instructing the user to consider how and why the anticipated impacts and implications will occur (see, for example, the instruction for part (b) of Section B2).

The tool aims to capture all types of impact, whether direct or indirect, intended or unintended, positive or negative (see the preamble to Section A5). It also aims to capture impacts that may occur at different levels, which in the case of HRH may include individual health workers, groups of health workers (e.g. a cadre) and employing organisations. Sections A5 and B2 of the draft HWIA tool aim to capture impacts of all relevant types and levels.

The draft HWIA tool aims to strike an appropriate balance between practicality and rigour and to facilitate a process that organisations involved in global health initiatives will find helpful, without being excessively bureaucratic and cumbersome. It therefore avoids complicated matrices and analysis techniques, instead using a mix of simple checklists in the screening module and open questions in the main module to obtain the relevant detail. This should encourage organisations involved in global health initiatives to build into each new initiative actions that will maximise the potential for positive HRH impacts and minimise the potential for negative ones. The tool does not require the user to provide quantitative data such as numbers of workers affected, although there is space for users to include these data if they are available and considered relevant. This is in recognition of the fact that the tool should be applicable to a wide range of types of initiative, and that there will usually be as much—if not more—interest in how and why the anticipated impacts will occur as in estimating the magnitude of the impact.

Requiring a full HWIA for every single grant, investment or resolution would be a heavy administrative burden, which would be a disincentive to carrying out an IA even when one would be helpful. The two-stage approach involves a relatively short and simple screening module (part A) to be completed for all grants, investments and resolutions, and a requirement to complete a full assessment (part B) only for those expected to have HRH implications or impacts.

Sometimes it will be clear that an initiative has HRH implications or impacts, but there will also be situations when the relevance of an initiative to the health workforce is less obvious, and situations in which there is a risk of unforeseen negative consequences for the health workforce. In such cases, IAs will help organisations to ensure that the impacts on and implications for HRH are properly taken into account [38], and may assist with ‘mainstreaming’ something that may otherwise be systematically overlooked [16, 37].

If carried out thoroughly, a full HWIA will be a time-consuming exercise. This brings with it a risk that people or organisations will be tempted to avoid a full assessment by indicating in the screening module that no HRH implications or impacts are anticipated. Similarly, there may be a temptation to manipulate the assessment to validate a personal preference, or a reluctance to give an honest opinion due to a negative organisational culture or fear of offending a donor. To mitigate against this, it is important to make the process and findings available for public scrutiny and debate [33–35]. Thus, if an assessment concludes that there will be no significant impact on or implications for HRH, readers and users of the completed tool or assessment report will either be able to satisfy themselves that this is a reasonable conclusion, or to challenge it in an informed way [15].

## Limitations

Because materials relevant to this analysis are not systematically captured in the peer-reviewed literature, but are rather scattered across a variety of different sources, and probably in some cases not publicly available at all, a key limitation of this method is that it may not have located all relevant examples of documentation relating to ex ante IA tools and techniques. However, as the identification of tools and other documents was designed to be illustrative rather than systematic, the approach taken was adequate to generate insights to inform the design of an HWIA tool. Due to the search being conducted in English, relevant examples in other languages were not captured, except for one item from Switzerland which was in French [22].

## Conclusion

To fulfil the WHA resolution relating to the Global Strategy on HRH, organisations involved in developing resolutions, strategic documents, grants and investments in support of global health objectives need to be able to carry out high-quality HWIAs. Based on an established HRH framework and good practice principles from other fields, a draft HWIA tool has been developed and is annexed to this paper. Following finalisation and adoption of the tool, it will be important to assess its use and any measurable effects it might have on improved design and/or impact on HRH.

## Additional file

Additional file 1: Supplementary annex: draft tool. (DOCX 43 kb)

## Abbreviations

EU: European Union
HRH: Human resources for health
HWIA: Health workforce impact assessment
IA: Impact assessment
IAIA: International Association for Impact Assessment
ILO: International Labour Organization
OECD: Organisation for Economic Cooperation and Development
UN: United Nations
UNFPA: United Nations Population Fund
UNICEF: United Nations International Children’s Emergency Fund
URL: Uniform resource locator (web address)
USAID: United States Agency for International Development
WHA: World Health Assembly
WHO: World Health Organization
