# Supplementary material for: Assessing the health workforce implications of health policy and programming: how a review of grey literature informed the development of a new impact assessment tool
Source: Hum Resour Health. 2017 Nov 9;15:79. doi: 10.1186/s12960-017-0252-x (PMC5679323; doi:10.1186/s12960-017-0252-x)
Supplement: Additional file 1: — Supplementary annex: draft tool. (DOCX 43 kb) [file 12960_2017_252_MOESM1_ESM.docx]

**Health Workforce Impact Assessment (HWIA) Tool**

**Introduction**

*Background*: Resolution WHA69.19 on the adoption of the WHO’s *Global Strategy on Human Resources for Health: Workforce 2030*, which was adopted by the 69^th^ World Health Assembly in May 2016, includes three key actions related to assessing the health workforce implications of health policy and programmes:

- Development partners, including bilateral partners and multilateral aid mechanisms, will augment, coordinate and align their investments in education, employment, health, gender and labour in support of domestic financing aimed at addressing national health workforce priorities,
- Global health initiatives will ensure that all grants include an assessment of health workforce implications, leverage national coordination and leadership, and contribute to efficient investment in and effective implementation of national health workforce policies
- An assessment of the health workforce implications will be made for technical resolutions brought before the World Health Assembly and WHO regional committees

This Health Workforce Impact Assessment (HWIA) tool has been designed by WHO, to provide a suggested structure for these assessments.

WHO defines the health workforce as “all people engaged in actions whose primary intent is to enhance health”. This includes **both health workers with clinical responsibilitie**s (e.g. doctors, nurses, midwives, pharmacists, community health workers) **and those who support their work** (e.g. health service managers, administrative workers, laboratory technicians, social workers, environmental health workers, and public health workers). For the purposes of this assessment, you should consider **all health workers, both public sector and private sector**.

*Who should use this tool*: This tool is designed for use by development partners and organisations involved in global health initiatives. It should be used whenever there is a plan to fund a health initiative via investments or grants, and every time a technical resolution is being prepared for consideration by the World Health Assembly or a WHO regional committee, **even if there are no immediately obvious health workforce implications**.

*When to complete this tool*: The form should be completed while the investment, grant or technical resolution is at the design stage, so that the findings of the assessment can be taken into account before critical decisions are made.

*How to complete this tool*: The tool is in two parts. Part A is a screening module, which prompts users to consider the ways in which the initiative will or might have health workforce implications. Completion of Part B is necessary only for initiatives with health workforce implications; it is a more detailed examination of these implications and how they can be addressed for successful implementation of the investment or resolution.

The tool consists of a mixture of questions requiring either a ‘check box’ response or a free text response. Questions are presented in tabular format, usually with the question wording in the left-hand column and space for the response in the right-hand column. For some questions in Part B, you will be prompted to provide evidence to support the responses given. If no such evidence is available, it is recommended that supporting evidence be gathered as part of the process of completing the HWIA. It is also recommended that the completed form be made publicly available.

**Process to conduct an impact screening of WHO technical resolutions and strategies**

The modality and process to apply this tool will vary according to the governance mechanisms of each organization. This section provides details on use for the screening and assessment of technical resolutions and strategies brought before the World Health Assembly or WHO Regional Committees.

*The department or unit initiating the development of a resolution/ strategy* should fill in as a matter of routine part A (screening) of this document when developing the draft resolution. The most appropriate timing would be when the resolution/ strategy is at a sufficiently advanced stage to assess its contents, but when it is still possible to make amendments to the text (should the outcome of the screening identify such a requirement).

*The department or unit initiating the development of a resolution/ strategy* submits the form, together with the resolution or strategy that it refers to, to the the health workforce department (for documents submitted to the WHA) or the health workforce regional focal point (for documents submitted to the Regional Committees).

*The health workforce department or the health workforce regional focal point* determines based on the responses to part A whether a full assessment is warranted. If no further assessment is warranted, the resolution/ strategy can proceed as per normal processes, and its submission to Governing Bodies is accompanied by a standard statement that a health workforce impact screening was conducted, and that this did not reveal any specific issue requiring particular consideration.

*If a further assessment is warranted, the health workforce department or the health workforce regional focal point* initiates a request for a face to face meeting with the originating department to conduct an interview aiming to fill in part B (full assessment). It is recommended that the initial meeting between the health workforce department or regional focal point and the originating department lasts approximately 1 hour. Following the meeting, the originating department fills in part B of the form, and the health workforce department or focal point assess it. Further meetings may be organized if agreed by both parties.

*The full health workforce impact assessment through part B of the form can result in 3 possible outcomes*:

1. no specific issue requiring particular consideration; consideration of the resolution/ strategy can proceed as per normal processes. Its submission to Governing Bodies is accompanied by a standard statement that a full health workforce impact assessment was conducted, and that this did not reveal any specific issue requiring particular consideration.
2. some specific modifications to the resolution or strategy are required to adequately factor in health workforce implications and requirements; once these are executed, consideration of the resolution/ strategy can proceed as per normal processes. Its submission to Governing Bodies is accompanied by a statement that a full health workforce impact assessment was conducted, and that the issues it revealed are fully addressed within the text of the resolution/ strategy.
3. the resolution or strategy has major workforce implications or requirements, which need to be factored in as part of the official process of consideration by Member States. An accompanying assessment report is developed by the health workforce department and annexed to the resolution/ strategy submitted by Governing Bodies to the Delegations of Member States.

**Part A: Screening**

| **Section A1: Details of the resolution or strategy being assessed** | | |
| --- | --- | --- |
| *No.* | *Question* | *Response* |
| A101 | **Name of technical resolution or strategy**, or summary description if it does not yet have a name. |  |
| A102 | **Region(s), country(ies) or sub-national area(s) to be targeted by this initiative.** If it is a global initiative, enter ‘global’. |  |
| A103 | **Which global, regional, national or sub-national human resources for health policies or strategies does it support?** | [List all relevant HRH policies and strategies and briefly explain how this initiative supports each one. If none, write ‘none’.] |
| A104 | **Reference number for technical resolution** (if applicable). |  |
| A105 | **Date this assessment was concluded** (month and year). |  |

| **Section A2: Contributors to the assessment** | | | | |
| --- | --- | --- | --- | --- |
| A201 | **Please provide the details of the person who led the process of completing this form** | | | |
| **Name** | | **Job title** | **Organisation** | **Email address** |
|  | |  |  |  |
| A202 | **Please provide the details of all other contributors to this assessment.** Add new rows to the table if necessary. | | | |
| **Name** | | **Job title** | **Organisation** | **Email address** |
|  | |  |  |  |
|  | |  |  |  |

| **Section A3: Objectives of the resolution or strategy** | | |
| --- | --- | --- |
| *No.* | *Question* | *Response* |
| A301 | **What problem(s) is the resolution trying to solve, or what situation is it trying to improve?** Describe in your own words. | [*Briefly describe the problem or situation, its scale, its causes and consequences, who it affects, how likely it is to persist in the absence of (further) intervention*.] |
| A302 | **Please list the specific objective(s) of the resolution and how these will be achieved**. Describe in your own words. | [*Briefly describe the objectives, and the inputs, processes and mechanisms that will be used to achieve them*.] |

| **Section A4: Extent to which successful implementation is dependent on the health workforce** | | | |
| --- | --- | --- | --- |
| *No.* | *Question* | *Response* | |
| **Is the successful achievement of the objectives listed at question A302 in any way dependent on …**  Select one answer for each question | | **Yes (definitely or probably)** | **No (definitely or probably not)** |
| A401 | **… enough health workers being available?** |  |  |
| A402 | **… people being able to access a health worker when they need to? (e.g. be able to travel to a health facility and/or pay the associated costs)** |  |  |
| A403 | **… people trusting in or finding acceptable the available health workers?** |  |  |
| A404 | **… the available health workers being motivated to provide particular services, having particular skills and/or having access to particular supplies or equipment?** |  |  |
| A405 | **… any other aspect(s) of the health workforce? (specify)** |  |  |

| **Section A5: Anticipated health workforce impacts** | | | |
| --- | --- | --- | --- |
| **In this section we ask you to anticipate possible direct and indirect impacts on the health workforce of the activities described at question A302. Impacts can be positive or negative, intended or unintended. They may affect the whole health workforce or just parts of it.**  **A direct impact occurs reasonably quickly after the action is taken, and it is clear that the action caused it to happen. An indirect impact may occur some time later, and may be caused by a direct impact of the action rather than by the action itself. For example, an action to train a cadre of health worker to perform a specific intervention may have the direct impact of improvements to health worker skills or competencies, and the indirect impact of increased respect for that cadre of health worker.**  **When answering questions in this section, focus exclusively on impacts on the health workforce. Other anticipated impacts - such as greater levels of coverage of key health interventions or improved health outcomes – are not relevant to this assessment.** | | | |
| *No.* | *Question* | *Response* | |
| **Which of the following things will happen as a direct or indirect result of the activities described at question A302?** Please consider both public and private sector health workers. | | **Yes (definitely or probably)** | **No (definitely or probably not)** |
| *Health labour market dynamics* | | | |
| A501 | **The creation of new jobs, preservation of existing jobs or filling of existing vacancies in the health sector** |  |  |
| A502 | **Job losses in the health sector, e.g. redundancies, phasing out of specific cadres of health worker** |  |  |
| A503 | **A change in the number of health workers choosing to leave the country or leave the health sector** |  |  |
|  | | **Yes (definitely or probably)** | **No (definitely or probably not)** |
| A504 | **A change in the proportion of health worker time spent providing services through the private sector** |  |  |
| A505 | **A change in the level of demand for the services of some or all health workers** |  |  |
| A506 | **A change in the level of domestic spending on the health workforce** |  |  |
| A507 | **A change in the profile of the health workforce (e.g. greater or lesser representation of ethnic minorities, refugees, women, disabled people)** |  |  |
| *Management and regulation of the health workforce* | | | |
| A508 | **Changed terms and conditions for health workers (e.g. responsibilities, hours of work, remuneration)** |  |  |
| A509 | **A change in the number of clinical interventions that health workers are authorised to perform** |  |  |
| A510 | **A change to the way in which all or some health workers are managed (e.g. introduction of new supervision arrangements, performance management techniques)** |  |  |
| A511 | **A change in the level of productivity or efficiency of all or some health workers** |  |  |
| A512 | **Increased or reduced administrative burden on health workers** |  |  |
| *Health worker education and training* | | | |
| A513 | **A change in the number of clinical interventions that health workers are competent to perform** |  |  |
| *Attitudes of and towards health workers* | | | |
| A514 | **A change in health workers’ confidence in their own abilities** |  |  |
| A515 | **A change in levels of health worker morale or motivation** |  |  |
| A516 | **A change in the way that health workers behave towards service users** |  |  |
| A517 | **A change in attitudes towards health workers, either from the general public or from their colleagues** |  |  |
| *Other* | | | |
| A518 | **Other anticipated impact(s) on the health workforce (specify)** |  |  |

**Part B: Full assessment**

| **Section B1: Understanding the initiative’s dependency on the health workforce** | | | | | |
| --- | --- | --- | --- | --- | --- |
| **Listed below are the options from Section A4, which assessed the extent to which this initiative is dependent on the health workforce. Refer back to Section A4, and select ‘yes’ in column (a) if you selected ‘yes’ in Section A4 and ‘no’ in column (a) if you selected ‘no’ in Section A4. Answer the questions in columns (b) (c) and (d) only if you select ‘yes’ in column (a).** | | | | | |
|  | Successful implementation is dependent on… | **(a) Response in Section A4** | **(b) Describe how and why the initiative is dependent on this, and to which types and cadres of health workers it applies. Obtain evidence to support the response.** | **(c) Are there plans to address this issue?** | **(d) If plans are in place to address this issue, describe these plans and obtain evidence if available. If not, what plans could be put in place?** |
| B101 | Enough health workers being available | Yes ⇨  No ⇩ |  | Yes ⇨  No ⇨ |  |
| B102 | People being able to access a health worker when they need to | Yes ⇨  No ⇩ |  | Yes ⇨  No ⇨ |  |
| B103 | People trusting in or finding acceptable the available health workers | Yes ⇨  No ⇩ |  | Yes ⇨  No ⇨ |  |
| B104 | Health workers being motivated or incentivised to provide particular services, having particular skills and/or having access to particular supplies or equipment | Yes ⇨  No ⇩ |  | Yes ⇨  No ⇨ |  |
| B105 | Other aspect(s) of the health workforce (specify) | Yes ⇨  No ⇩ |  | Yes ⇨  No ⇨ |  |

| **Section B2: Understanding the initiative’s impact on the health workforce** | | | | | |
| --- | --- | --- | --- | --- | --- |
| **Listed below are the section headings from Section A5, which assessed possible impacts on the health workforce. Refer back to Section A5, and if you answered ‘yes’ to any of the questions under that heading, select ‘yes’ in column (a). If you answered ‘no’ to all of the questions under that section heading select ‘no’ in Column (a). Answer the questions in columns (b) (c) and (d) only if you select ‘yes’ in column (a).** | | | | | |
|  | Possible health workforce impacts in relation to… | **(a) Was ‘yes’ selected for any of these questions?** | **(b) Describe the nature of the impact(s), explain which types and cadres of health workers will or might be affected, and explain how and why these impacts will or might occur. Obtain supporting evidence.** | **(c) Are there plans to make positive impacts more likely or protect against negative impacts?** | **(d) If plans are in place, please describe these plans and obtain evidence. If not, what plans could be put in place to make positive impacts more likely and protect against negative impacts?** |
| B201 | Health labour market dynamics (questions A501 to A507) | Yes ⇨  No ⇩ |  | Yes ⇨  No ⇨ |  |
| B202 | Management and regulation of the health workforce (questions A508 to A512) | Yes ⇨  No ⇩ |  | Yes ⇨  No ⇨ |  |
| B203 | Health worker education and training (question A513) | Yes ⇨  No ⇩ |  | Yes ⇨  No ⇨ |  |
| B204 | Attitudes of and towards health workers (questions A514 to A517 | Yes ⇨  No ⇩ |  | Yes ⇨  No ⇨ |  |
| B205 | Other health workforce impacts (question A518) | Yes ⇨  No ⇩ |  | Yes ⇨  No ⇨ |  |

| **Section B3: Equity analysis** | | | |
| --- | --- | --- | --- |
| **Is it possible that this initiative will affect some health workers more than others because of …** | | **(a) Because of this, some groups of health worker will or might…** select all that apply | **(b) Explain which groups will or might be affected, how and why. State what will or could be done to minimise the risk of creating or widening inequity. Obtain supporting evidence.** |
| B301 | **… their personal characteristics, e.g. sex, age, marital status, parental status, race, ethnic group, religion or belief, language?** | Benefit  Be disadvantaged  Neither |  |
| B302 | **… their geographical location?** | Benefit  Be disadvantaged  Neither |  |
| B303 | **… their employment status (e.g. permanent/temporary, full/part time)** | Benefit  Be disadvantaged  Neither |  |
| B304 | **… their employer (e.g. public sector, private sector)** | Benefit  Be disadvantaged  Neither |  |
| B305 | **Other (specify)** | Benefit  Be disadvantaged  Neither |  |

| **Section B4: Stakeholder analysis** | | | | | |
| --- | --- | --- | --- | --- | --- |
| B401 | **In column (a), make a list of stakeholders, then for each one, answer the questions in columns (b), (c), (d) and (e). Please consider all health workforce stakeholders who will or might have an interest in this initiative. Stakeholders may include: national governments, local governments, individual parliamentarians, international organisations, professional associations, NGOs, private sector providers, donors, academics, technical experts, CSOs and others.**  **There is space for four stakeholders; add more rows if needed.** | | | | |
| **(a) Name of stakeholder** | | **(b) Nature of their interest** (select all that apply) | **(c) Likely attitude** | **(d) Level of influence** | **(e) Comments (highlight most important stakeholders, describe how relationships will be managed)** |
|  | | They will or might be influential  They will or might be affected  Interested party | Positive  Neutral  Negative | High  Low |  |
|  | | They will or might be influential  They will or might be affected  Interested party | Positive  Neutral  Negative | High  Low |  |
|  | | They will or might be influential  They will or might be affected  Interested party | Positive  Neutral  Negative | High  Low |  |
|  | | They will or might be influential  They will or might be affected  Interested party | Positive  Neutral  Negative | High  Low |  |

| **Section B5: Legal and political considerations** | | | |
| --- | --- | --- | --- |
| *No.* | *Question* | *Response* | |
| B501 | **Will the implementation of this resolution require compliance with any national or international employment laws, guidelines or regulations in respect of the health workforce?** | Yes  No | [*If yes, list the relevant law(s), guideline(s) or regulation(s) and state how compliance will be assured. If no, explain why compliance is not relevant*.] |
| B502 | **Might the HRH implications be politically controversial?** | Yes  No | [*If yes, explain the reason for the (potential) controversy, what effect(s) this may have, and how it will or could be managed. If no, explain why not*.] |

| **Section B6: Next steps** | | | | |
| --- | --- | --- | --- | --- |
| B601 | **Has this assessment highlighted any HRH impacts or implications that require additional attention before the resolution or strategy is finalised?** | | Yes ⇨ complete grid below (add more rows if needed)  No ⇨ go to Section B7 | |
| **Impact or implication requiring additional attention** | | **Action(s) required to address this** | **Responsible person(s) or organisation(s)** | **Review date** |
|  | |  |  |  |
|  | |  |  |  |
|  | |  |  |  |
|  | |  |  |  |
| **Section B7: Additional comments** | | | | |
| B701 | **Please use the space below to record any additional issues or comments that are relevant to this assessment but were not covered above.** | | | |
|  | | | | |

**Glossary**

| Cadre | A group of health workers specifically trained for a particular purpose or type of work. |
| --- | --- |
| Clinical | Relating to the observation and treatment of patients (rather than theoretical or laboratory work) |
| Competency | Definition of the requirements for success in a particular task or job |
| CSO | Civil Society Organisation |
| Efficiency | The accomplishment of something without wasted time and effort |
| Equity | Fairness and impartiality |
| Focal area(s) | The region(s), country(ies) or sub-national area(s) targeted by the investment, grant or technical resolution |
| Health workforce / human resources for health | All people engaged in actions whose primary intent is to enhance health, whether they work in the public sector or the private sector. This includes both health workers with clinical responsibilities (e.g. doctors, nurses, midwives, pharmacists, community health workers) and those who support their work (e.g. health service managers, administrative workers, laboratory technicians, social workers, environmental health workers, public health workers) |
| NGO | Non-Governmental Organisation |
| Out-of-pocket spending | Costs of goods or services that people pay for directly out of their own cash reserves, rather than paying for them indirectly via taxation or insurance |
| Private sector | The part of the national economy that is not under direct state control. In the health sector this can include private companies, NGOs, faith-based organisations etc |
| Productivity | A measure of the efficiency of a person, system, machine etc. Typically estimated by dividing output by resources used. |
| Remuneration | Reward for employment, including wages, salary, pay, allowances, bonuses, incentives and benefits (e.g. health insurance, pension contributions) |
| Skill | A specific learned activity |
| Voluntary attrition | A decision made by an employee to leave a job before reaching statutory retirement age |
